# Fluorescence Sensing of the Interaction between Biomembranes with Different Lipid Composition and Endocrine Disrupting Chemicals

**DOI:** 10.3390/ma7010170

**Published:** 2013-12-31

**Authors:** Yuko Nakane, Izumi Kubo

**Affiliations:** 1 Quantitative Biology Center, RIKEN, 6-2-3 Furuedai, Suita, Osaka 565-0874, Japan; E-Mail: ynakane@riken.jp; 2 Department of Bioinformatics, Faculty of Engineering, Soka University, 1-236 Tangi, Hachioji, Tokyo 192-8577, Japan

**Keywords:** fluorescence sensing, endocrine disrupting chemicals, biomembrane, liposomes, pattern analysis

## Abstract

Fluorescence sensing of the interaction between biomembranes with different lipid composition and endocrine disrupting chemicals (EDCs) was carried out by using a liposome-encapsulating fluorescence dye (carboxyfluorescein (CF)-liposome). We detected a significant increase in fluorescence intensity in CF-liposome solutions due to the leakage of fluorescence caused by the interaction of EDCs with the biomembranes of liposomes. The temporal increases in fluorescent were significantly different among the lipid compositions of CF-liposome and the EDCs. Results were considered by summarizing the interactions in radar charts and by showing the pattern of interaction of each EDC. Each chart showed a dissimilar pattern reflecting the complexity of the biomembrane-EDC interaction. The results indicate that this fluorescence sensing could be useful to evaluate the interaction.

## 1. Introduction

Endocrine disrupting chemicals (EDCs) are organic chemicals that interfere with the endocrine system [1]. Recently, some reports based on *in vivo* and *in vitro* experiments have suggested that exposure to EDCs may cause adverse effects on sexually-dimorphic behaviors, reproduction, and neurodevelopment [2,3]. To assess harmful potential of EDCs on wildlife and human, it is important to evaluate the interaction between EDCs and biomembrane as the interaction and then passive distribution is one of transport route of EDCs from environment to an organism. Some EDCs, such as bisphenol A, have multi-pathway mediated transporters and receptors on the surface of cell membranes [4–8], and some EDCs are transported through active transport [9]. Electrostatic properties of some environmental chemicals are related to such transportation [10]. In addition, some EDCs are transported passively through biomembrane, by interaction between EDCs and the membrane. We have been investigating the interaction and related properties of EDCs have been examined, such as hydrophobic property and polarizability, to date [11]. In this study, we consider that the EDCs-biomembrane interactions should not be overlooked for a better understanding of the behavior of EDCs.

Langmuir-Blodgett film, the bilayer lipid membrane, and liposomes, are useful biomembrane models that have been used to evaluate the permeability of cell membranes to environmental organic pollutants, including structurally diverse EDCs [12–14]. Liposomes, bilayers of lipid vesicles, are an excellent model of biological membranes. They are convenient sensing materials due to their simple preparation, varied functionalization, and easy to handle.

Kwon *et al.* [15,16] showed that 20 structurally diverse EDCs permeated into the membrane of liposomes and evaluated the EDC’s permeability as liposome/water partition coefficient by using large unilamellar liposomes with dipalmitoylphosphatidylcholine (DPPC).

We have examined the penetration of EDCs into biomembrane models in order to detect EDCs by using a liposome-based membrane model [17–19]. In our previous work, we used liposome-encapsulating self-quenching carboxyfluoresceine (CF-liposome) and demonstrated that it could detect EDCs as there was an increase in fluorescence intensity caused by the induced release of the encapsulated carboxyfluorescein from the inside to the outside of the liposomes [11].

In this work, we applied the sensing principle to evaluate the interaction between EDCs and the biomembranes with different lipid composition, and to fulfill pattern analysis of EDCs by using CF-liposomes as the lipid compositions of biomembranes differ widely in biological species and organs [20]. Pattern analysis is useful to consider the complex analysis of groups, such as food, drinks, drugs, and environmental pollutants. Toko *et al.* [21,22] reported the interaction between various compositions of artificial lipid membranes and substances with different tastes (e.g., salty, sour, sweet, *etc.*), and its application to multichannel taste sensors. These sensors are as because they could quantify a basic taste without multivariate analysis. A similar method for the analysis of the interaction between a cell membrane model and environmental pollutants has never been reported.

**Figure 1.**
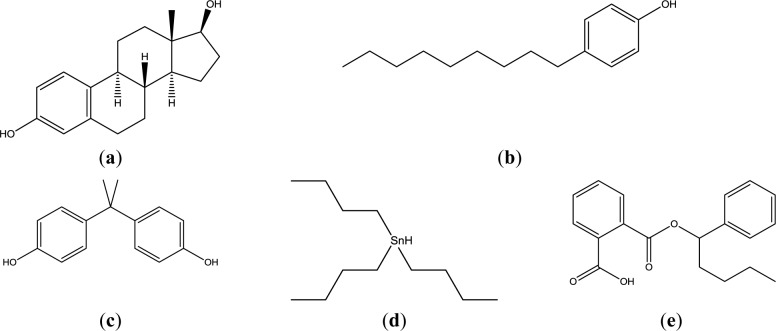
Chemical structure of natural hormone and EDCs. (**a**) Estradiol; (**b**) Nonylphenol; (**c**) bisphenol A; (**d**) tributyltin; and (**e**) benzylbutylphtalate.

Actual mammalian cell membrane is constituted of glycerophospholipids, sphingophospholipids, cholesterol, and so on. In these lipids, glycerophospholipids (mainly phosphatidylcholine and phosphatidylethanolamine), which are not a single molecular species, are main components. In this study, phosphatidyldholine and phosphatidlethanolamine was substituted by 1,2-dipalmitoyl-sn-glycero-3-phosphocholine (DPPC), and 1,2-dioleoyl-sn-glycero-3-phosphoethanolamine (DOPE) to understand the interaction simply. This lipid and cholesterol were examined as liposomes with four lipid compositions. Four EDCs, namely nonylphenol (NPh), bisphenol A (BPA), buthylbenzylphthalate (BBP), and tributyltin (TBT), were examined as they are widespread around the world in water and food chains, and because their chemical structures are not similar (Figure 1).

## 2. Results and Discussion

### 2.1. Fluorescence Changes of the CF-Liposomes

**Figure 2.**
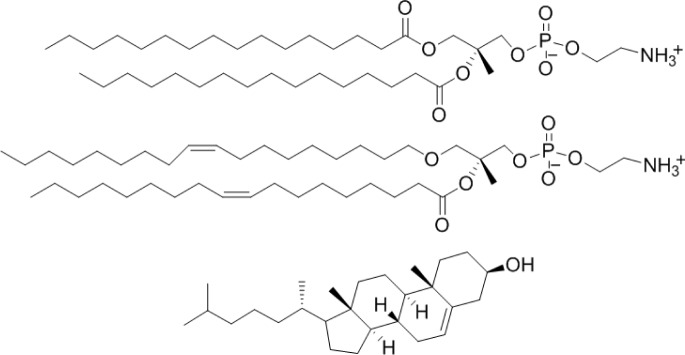
Chemical structures of lipids. DPPC (**top**); DOPC (**center**); and cholesterol (**bottom**).

Liposomes with four different lipids compositions were prepared; DPPC = 100% (DPPC-CF-liposome), DPPC:cholesterol = 80:20 mol% (PCch-CF-liposome), DPPC:DOPE = 50:50 mol% (PCPE-CF-liposome), and DPPC:DOPE:cholesterol = 40:40:20 mol% (PCPEch-CF-liposome) were prepared. The chemical structures of the lipids, DPPC, DOPE, and cholesterol are shown in Figure 2.

**Figure 3.**
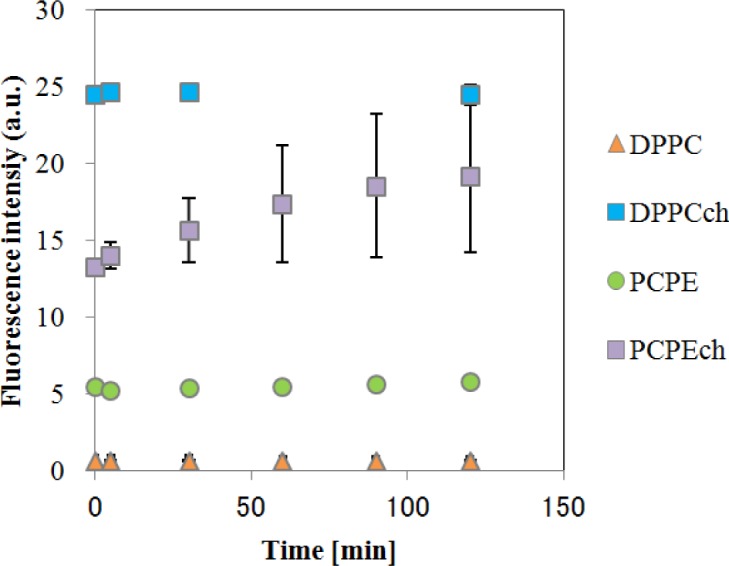
Temporal fluorescence changes of the CF-liposome. All of plots are an average of three samples. The error bars indicate standard deviation.

The temporal changes in fluorescence of CF-liposomes are shown in Figure 3. All liposomes, except for DPPC-CF-liposome, showed a slight increase in fluorescence intensity caused by natural leakage of fluorescence dye. As the increases were gradual, these natural leakages were corrected to consider the effect of EDCs on the liposome membrane. For the correction, fluorescence during the interaction between EDCs and liposomes (F_i_) were divided by the fluorescence of natural leakage (F_n_) and F_i_/F_n_ was defined as F. The fluorescence intensity of CF-liposomes ruptured forcibly by 0.1% (v/v) Triton-X 100 was used as the standard (F_T_) for normalization as each measurement for the CF-liposomes with each lipid composition was carried out on an individual microplate.

### 2.2. Interactions of EDCs or the Natural Hormone to Biomembrane

The interaction of EDCs or the natural hormone to biomembrane (*I*_F_), measured by fluorescence, was calculated according to Equation (1):

$$I_{F}=F/F_{T}$$ (1)

**Figure 4.**
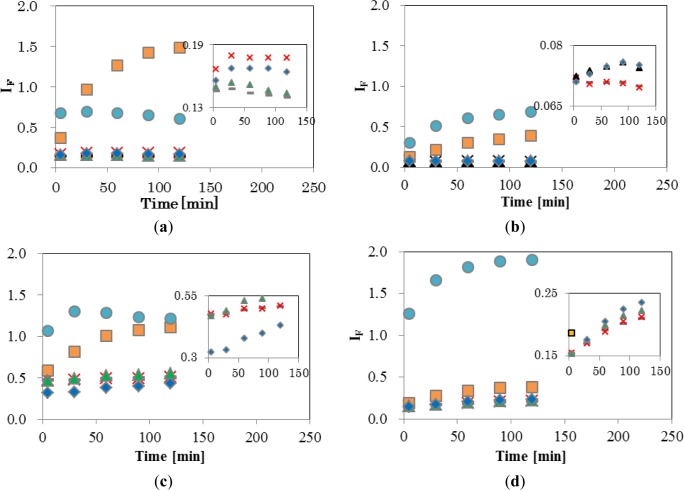
Temporal changes of I_F_. (**a**) DPPC-CF-liposome; (**b**) DPPCch-CF-liposome; (**c**) PCPE-CF-liposome; and (**d**) PCPEch-CF-liposome. – (gray); negative control, × (red); E2, □ (orange); NPh, ▲ (green); BPA, ⋄ (blue); BBP and ● (light blue); TBT. Sample size; *n* = 3, standard deviations are 10% or less.

Figure 4 shows the temporal changes in I_F_ of the four EDCs and natural hormone (β-estradiol; E2), while the inset shows an enlargement of the range of low I_F_. The final concentrations of EDCs and E2 were 5 ppm (mg/L). As shown in Figure 4a, DPPC-CF-liposome, *I*_F_ of NPh, and TBT, increased significantly. A gradual increase of *I*_F_ by NPh was observed during 120 min, finally reaching 1.48. On the other hand, the *I*_F_ of TBT increased quickly and reached a plateau after 5 min of interaction with TBT. These different patterns of increase between NPh and TBT indicate that these molecules behave differently in the membrane. A comparison of the chemical structure between NPh and TBT supports the differences in increasing patterns. As the NPh molecule has a polar group, an alkyl chain and has a rod-like shape, it might penetrate into the DPPC membrane by a hydrophobic interaction between the membrane and the alkyl chain of NPh, and wander among the lipid molecules in the membrane, thus, bringing about an increase in membrane fluidity. The gradual release of CF from liposomes continued over 120 min. On the other hand, the TBT molecule has three non-polar butyl groups and has a plane-like shape. Thus, it was considered that TBT molecules penetrate into the DPPC membrane rapidly with a leakage of CF and then fix to the surrounding lipid molecules by a hydrophobic interaction.

E2, BPA, and BBP showed low *I*_F_ of DPPC-CF-liposome (Figure 4a inset). These chemicals were reported to undergo the pathway, mediated with transporters and receptors on the surface of cell membrane.

Figure 4b shows a plot of the DPPCch-CF-liposome. All values of I_F_, except for NPh, were lower than I_F_ values of DPPC-CF-liposome. Cholesterol in the CF-liposome may have stabilized the membrane and the stable membrane may, in turn, have prevented the leakage of dye from liposomes after interacting with EDCs.

Figure 4c shows the I_F_ of PCPE-CF-liposome. Compared to DPPC-CF-liposome (Figure 4a), all values of I_F_, except for NPh, were higher. The increase in I_F_ might be occurred by phase effect and an increase in membrane fluidity. As the phase transition temperature (*T*_c_) of DPPC and DOPE are 41 °C and −18 °C, respectively, the membrane of PCPE-CF-liposome were mixture of gel phase DPPC lipids and liquid crystalline phase (L_c_) DOPC lipids. The addition of the L_c_ phase lipids in the membrane make the membrane more fluidic than that of DPPC-CF-liposome.

Figure 4d shows the plot of PCPEch-CF-liposome. The values of I_F_, except for TBT, were lower than that of PCPE-CF-liposome (Figure 4c), but higher than that of DPPCch-CF-liposome (Figure 4b). The effect of cholesterol to the interaction between liposome and EDCs was consistent in PCPE membrane and DPPC membrane.

These results indicate that changes in membrane fluidity, including the phase effect after the interaction with EDCs, is one of the most effective properties of I_F_. However, there are likely to be unknown properties of I_F_ as NPh and TBT behaved differently to other EDCs. The chemical structure of TBT is significantly different from the structure of other EDCs. TBT is hydrophobic and has three flexible alkyl chains, the latter supposedly being easier to penetrate into the inside of the bilayer lipid membrane of liposomes than other EDCs, and change the fluidic properties of the membrane through their hydrophobic interaction with surrounding lipid molecules. Thus, I_F_ reflects the complex interaction through a combination of the property of lipid, the lipid composition, and the chemical property of EDCs. Although it is important to elucidate the factors and mechanisms causing the change in I_F_, the method used to evaluate for this complex interaction is also important.

As the lowest EDCs concentration of the appearance of biological effect in human breast cancer and trophoblast cells was 1 μM [23–26], it was reasonable that the interaction in the present study occurred at 5 ppm (NPh; 22 μM, BPA; 22 μM, BBP; 16 μM, TBT and 17 μM). As a large increase of I_F_ was observed in NPh and TBT, this sensing method would have the potential to detect the interaction at the lower concentration of EDCs.

### 2.3. Pattern Analysis of EDCs with CF-Liposomes

**Figure 5.**
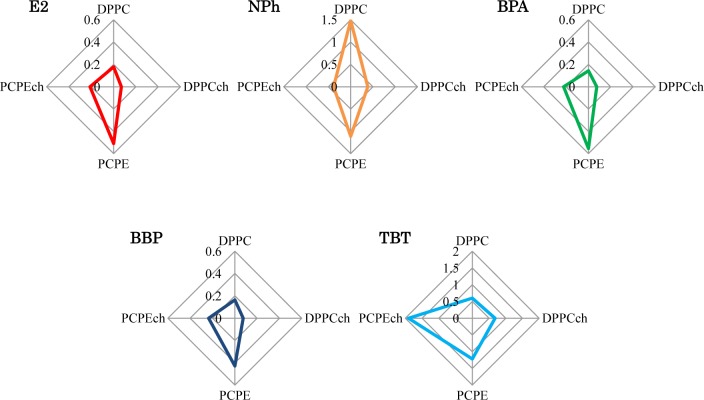
Radar charts of I_F_ of EDCs and E2. The I_F_ values at 120 min in Figure 4 are plotted.

The liposomes with different lipid composition showed varying interactions after exposure to four EDCs. To consider these complex interactions, the I_F_ values after 120 min were summarized in radar charts (Figure 5). Interestingly, no EDC showed the same result. Although the charts of the chemicals (E2, BPA, and BBP), which have the pathways different from the interaction with lipid membrane in cell membrane, were similar, they were not identical. These dissimilar patterns imply the presence of effective factors caused by the chemical properties of each EDCs and/or the interaction between EDCs and membranes. These complex interactions are likely to be an obstacle in our understanding of EDCs. In general, different animal species, such as fish or mammals, and their organs, such as brain or liver, are composed of different lipids. This differentiation indicated the importance of the evaluation of the interaction between the membrane composed with various lipid and EDCs. Although in the human body, temperature and pH are maintained constant by homeostasis, the interaction also might be affected by the environmental physico-chemical condition, such as temperature and pH. Temperature change causes lipids phase transition and pH change shift the chemical property of EDCs, such as solubility of water [27] and liposome-water distribution ratio [28]. Our results in the chart suggest the necessary of the global evaluation of membrane-EDCs interaction.

## 3. Experimental Section

### 3.1. Materials

DPPC, cholesterol and 5(6)-carboxyfluorescein (CF) was obtained from Sigma-Aldrich Co. Ltd., Tokyo, Japan. DOPE was purchased from Avanti Polar Lipid, Inc. NPh and BPA were purchased from Kanto Chemical Co., Inc. BBP was obtained from Wako Pure Chemical Industries, Ltd., Osaka, Japan. NPh, BPA, BBP, and TBT were of laboratory grade and were used without further purification.

Water used in this study was purified to 18.3 MΩ by a Mill Q SP system.

### 3.2. Liposome Preparation

Liposomes were prepared according our previous report [11]. The lipids were dissolved in a glass vial by 1.5 mL organic solvent (chloroform:methanol = 90%:10%, v/v). The final concentration of total lipids, including cholesterol, was 26.7 mM. The solvent was removed by nitrogen gas, and completely evaporated in a desiccator overnight. Calboxyfluorescein (CF) solution (1.5 mL of 0.15 M CF in 10 mM phosphate buffer) was added to hydrate the lipid films by incubating at the above *T*_c_ overnight. Then the solution was sonicated with a microchip sonicator for 10 min. Immediately after sonication, the liposome suspension was filtered several times through a polycarbonate nucleopore membrane with a 0.4 μm pore size and then re-filtrated twice through the same type of membrane with a 0.2 μm pore size. The prepared liposomes were smaller than 0.2 μm in a diameter. The liposomes encapsulating CF were separated from free CF by centrifugation at 30,000 rpm, 4 °C, for 30 min after dilution of the external solution with water. The precipitated liposomes were resuspended in 2 mL of 10 mM phosphate buffer solution (PB) at pH 7.0. The suspension was stored at 4 °C.

### 3.3. Fluorometric Analysis

The CF-liposome suspensions were prepared in 1 mL of PB. The prepared suspension was diluted to 33.4 μM in a final lipid concentration with PB. As the high concentration of the encapsulated dye led to self-quenching, the resulting fluorescence intensity of the liposome suspension was very weak. To evaluate the interaction of each chemical to liposomes, 200 μL of each liposome was mixed with 5 ppm (5 mg/L) of EDCs in a 96-well micro plate (Corning) and kept at 25 °C for 120 min. Fluorescence intensity was measured with a microfluorometer (Fluoreskan Ascent, Thermo Fisher Scientific K.K., Yokohama, Japan) at an exciting wavelength of 490 nm and emission wavelength of 515 nm, 5 min after mixing with EDCs and then at 30, 60, 90, and 120 min.

## 4. Conclusions

We prepared four types of lipid composition liposomes to investigate the interaction between biomembranes with these different lipid compositions and EDCs with dissimilar chemical structures. All the prepared liposomes that encapsulated self-quenching fluorescence dye showed an increase in fluorescence intensity after exposure to the EDCs. Differences of the temporal increase indicated that the interaction between biomembrane and the chemical nature of EDCs depend on not only the membrane fluidity of lipids but also other potential factors. We summarized the differentiation of the interactions in radar charts. Each chart showed dissimilar pattern considered due to the complexity biomembrane-EDCs interaction occurred by preceding multi-factors. This is the first report of fluorescence-based sensing of the different interaction between the different lipid composition biomembranes and EDCs. Owing to their easy preparation and handling, liposomes are friendly biomaterials to combine high-throughput analysis systems, such as a micro electro mechanical systems technique. Thus, this fluorescence sensing, by using CF-liposomes, is expected to apply the global pattern analysis for the interaction between various lipid composition biomembrane.
